# Antigenic characterization of SARS-CoV-2 Omicron subvariant BA.4.6

**DOI:** 10.1038/s41421-022-00493-0

**Published:** 2022-11-29

**Authors:** Aiste Dijokaite-Guraliuc, Raksha Das, Rungtiwa Nutalai, Daming Zhou, Alexander J. Mentzer, Chang Liu, Piyada Supasa, Susanna J. Dunachie, Teresa Lambe, Elizabeth E. Fry, Juthathip Mongkolsapaya, Jingshan Ren, Jiandong Huo, David I. Stuart, Gavin R. Screaton

**Affiliations:** 1 Wellcome Centre for Human Genetics, Nuffield Department of Medicine, University of Oxford, Oxford, UK; 2 Division of Structural Biology, Nuffield Department of Medicine, University of Oxford, The Wellcome Centre for Human Genetics, Oxford, UK; 3 Chinese Academy of Medical Science (CAMS) Oxford Institute (COI), University of Oxford, Oxford, UK; 4 Oxford University Hospitals NHS Foundation Trust, Oxford, UK; 5 Peter Medawar Building for Pathogen Research, Oxford, UK; 6 Centre For Tropical Medicine and Global Health, Nuffield Department of Medicine, University of Oxford, Oxford, UK; 7 Mahidol-Oxford Tropical Medicine Research Unit, Department of Medicine, University of Oxford, Oxford, UK; 8 Oxford Vaccine Group, Department of Paediatrics, University of Oxford, Oxford, UK; 9 State Key Laboratory of Respiratory Disease, National Clinical Research Center for Respiratory Disease, Guangzhou Institute of Respiratory Health, the First Affiliated Hospital of Guangzhou Medical University, Guangzhou, Guangdong, China; 10 Guangzhou Laboratory, Bio-island, Guangzhou, China; 11 Diamond Light Source Ltd, Harwell Science & Innovation Campus, Didcot, UK

**Keywords:** Biological techniques, Immunology

Dear Editor,

Since the emergence of the Omicron BA.1.1.529 variant of SARS-CoV-2 in November 2021^1^, a number of Omicron sublineages with increased antibody evasion capacity and transmissibility have been identified and caused regional and global outbreaks, including BA.1.1, BA.2, BA.2.12.1 and BA.4/5^2–4^. While BA.4 and BA.5 share identical spike (S) sequence, for unknown reasons BA.5 outcompeted BA.4 in many regions, with a global prevalence of 77.1% as of epidemiological week 40 (3rd–9th October 2022) (https://reliefweb.int/report/world/coronavirus-disease-covid-19-weekly-epidemiological-update-26-october-2022).

Recently, a new variant related to BA.4/5, designated BA.4.6, has emerged and expanded in the United States where BA.5 dominates [80.3% prevalence as of 31st October 2022 (https://cov-spectrum.org/explore/United%20States/AllSamples/from=2022-07-01&to=2022-11-01/variants?nextcladePangoLineage=ba.5*&)], rising from < 2% of sequences in early July to 11.7% as of 31st October 2022 (https://cov-spectrum.org/explore/United%20States/AllSamples/from=2022-07-01&to=2022-11-01/variants?nextcladePangoLineage=BA.4.6&). Compared to BA.4/5, BA.4.6 contains two extra mutations in the Spike protein (S), R346T in the Receptor Binding Domain (RBD) and N658S in the C-terminal domain. The R346T mutation has raised concern for enhanced antibody evasion over BA.4/5, as the R346K mutation in BA.1.1 reduced serum neutralization compared to BA.1 and impaired the activity of a number of monoclonal antibodies (mAbs)^2^. Here, we study the neutralization profile of BA.4.6 using Pfizer-BioNtech vaccine serum, BA.1, BA.2, and BA.4/5 vaccine breakthrough immune serum (characteristics of sample donors are shown in Supplementary Table S1), as well as panels of mAbs. Remarkably, we show further antibody evasion of BA.4.6, providing guidance for vaccine design and the use of therapeutic monoclonals.

To evaluate the antibody evasion capacity of BA.4.6, we constructed a panel of pseudotyped lentiviruses^5^ expressing the S gene from BA.4.6 and other SARS-CoV-2 variants together with early pandemic Wuhan-related strain, Victoria, used as a control. Firstly, we examined the neutralization profile with sera collected 4 weeks following a third dose of the Pfizer-BioNtech vaccine BNT162b2 (*n* = 22). Compared to BA.4/5, neutralization titers against BA.4.6 were reduced twofold (*P* < 0.0001) for BNT162b2 sera (Fig. 1a).

**Fig. 1:**
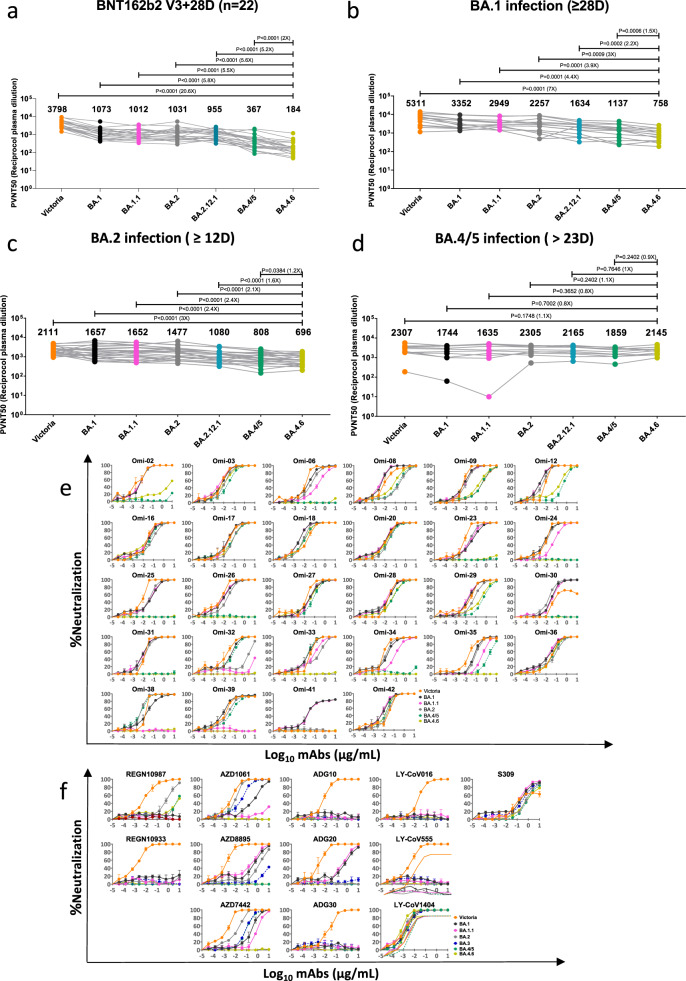
Characterisation of BA.4.6 by pseudoviral neutralization assay. **a**–**d** Pseudoviral neutralization assays of BA.4.6 by vaccine, BA.1, BA.2, and BA.4.5 immune serum. IC_50_ values for the indicated viruses using serum obtained from vaccinees 28 days following their third dose of Pfizer BNT162b2 vaccine (*n* = 22, **a**). IC_50_ values for the indicated viruses against serum from volunteers suffering vaccine breakthrough BA.1 (*n* = 14, **b**), BA.2 (*n* = 23, **c**) and BA.4/5 (*n* = 11, **d**) infections. Geometric mean titers are shown above each column. The Wilcoxon matched-pairs signed rank test was used for the analysis and two-tailed *P* values were calculated. **e** Neutralization curves for a panel of 28 monoclonal antibodies made from samples taken from vaccinees infected with BA.1 against BA.4.6 were compared with Victoria, BA.1, BA.1.1, BA.2, BA.4/5, and BA.2.75 variants. Error bars represent means ± SEM of repeat experiments. **f** Neutralization curves for a panel of 12 commercial monoclonal antibodies against same variants. IC_50_ ± SEM values are shown in Supplementary Table S2.

Next, we assayed the neutralization profile for serum samples collected from vaccinees infected with BA.1 [samples (*n* = 16), taken ≥ 28 days following symptom onset], BA.2 [samples (*n* = 23), taken ≥ 12 days following symptom onset] or BA.4/5 [samples (*n* = 11, all but one vaccinated), taken > 23 days following symptom onset] (Fig. 1b–d). Neutralization titers against BA.4.6 were significantly reduced compared to BA.4/5 for both breakthrough BA.1 (1.5-fold; *P* = 0.0006) and BA.2 (1.2-fold; *P* = 0.0384) serum samples. Notably, BA.4.6 was able to effectively escape neutralization by serum samples from BA.1 breakthrough infections, showing a substantial reduction in titers compared to BA.1 (4.4-fold; *P* = 0.0001), BA.2 (threefold; *P* = 0.0009) and BA.4/5 (1.5-fold; *P* = 0.0006). A small non-significant increase in neutralization titers against BA.4.6 was observed in the BA.4/5 breakthrough cohort compared to BA.4/5. Of note, the single serum sample from the unvaccinated BA.4/5 convalescent showed lower levels of neutralization to most variants, especially BA.1 and BA1.1. It is not clear why the BA.2 and BA.4/5 neutralization titers using BA.2 and BA.4/5 serum respectively were not higher than titers for other SARS-CoV-2 variants as one might expect.

To further characterize the antigenic escape properties of BA.4.6, we performed pseudoviral assays on a panel of potent human mAbs generated from BA.1 breakthrough convalescents^2^ (Fig. 1e). In general, the neutralization profiles of BA.4.6 were similar to those of BA.4/5. However, the residual activity of Omi-35 (IC_50_ = 1.687 µg/mL) was further knocked out for BA.4.6, and the potency of Omi-32 and Omi-33 against BA.4/5 (IC_50_ = 0.035 and 0.013 µg/mL, respectively) was completely impaired for BA.4.6. The loss in activity of Omi-32 could be explained by the disruption of the interaction between CDR-H1 and R346 by the R346T mutation, as illustrated by previous structural analysis^2^.

Finally, we evaluated the neutralization activities of a number of mAbs in clinical use (Fig. 1f). The potency of AZD1061/cilgavimab against BA.4/5 was completely knocked out against BA.4.6, leading to a total loss in activity of AZD7742/Evusheld (a combination of AZD1061/cilgavimab and AZD8895/tixagevimab which is already inactive against BA.4/5). The activity of S309/sotrovimab [no longer authorized by the U.S. food and drug administration (FDA) for COVID-19 treatment since April 2022 due to its inefficacy against BA.2] was further reduced compared to BA.2 and BA.4/5. This, therefore, leaves LY-CoV1404/bebtelovimab the only option for the treatment of BA.4.6.

In summary, BA.4.6 showed further reduction in neutralization by serum from triple dose Pfizer vaccinees, as well as from BA.1 and BA.2 vaccine breakthrough convalescents compared to BA.4/5, which is in line with recent reports^6^. Notably, BA.4.6 does not seem to be more resistant to neutralization by serum from BA.4/5 breakthrough infection compared to other variants. This altogether suggests that there is a strong likelihood of infection or breakthrough infection by BA.4.6 unless one has been triply vaccinated and recovered from BA.4/5 infections, which seems to provide some protection against BA.4.6. Mutation R346T has been acquired by a number of emerging SARS-CoV-2 strains, notably BA.7 a derivative of BA.5 which is increasing in a number of locations (https://cov-spectrum.org/explore/United%20Kingdom/AllSamples/Past6M/variants?nextcladePangoLineage=bf.7*&).

Bivalent booster vaccination, combining the ancestral strain with Omicron BA.1 is being rolled out in the UK (https://www.gov.uk/government/news/pfizerbiontech-bivalent-covid-19-booster-approved-by-uk-medicines-regulator), and has been recently authorized by FDA (https://www.fda.gov/news-events/press-announcements/coronavirus-covid-19-update-fda-authorizes-moderna-pfizer-biontech-bivalent-covid-19-vaccines-use). It remains to be seen how effective these bivalent boosters are at preventing BA.4.6 infection. Finally, BA.4.6 has further impaired the activity of Evusheld which remained active against BA.4/5; as a result, now only LY-CoV1404/bebtelovimab retains potency against all circulating SARS-CoV-2 variants.

## Supplementary information

Supplemental Material File #1

BA.4.6_Supplementary information
