# Supplementary material for: Antigenic characterization of SARS-CoV-2 Omicron subvariant BA.4.6
Source: Cell Discov. 2022 Nov 29;8:127. doi: 10.1038/s41421-022-00493-0 (PMC9705283; doi:10.1038/s41421-022-00493-0)
Supplement: Supplementary file 1 — Supplemental Material File #1 [file 41421_2022_493_MOESM1_ESM.pdf]

Ethics approval and consent to participate

Sera from BA.1 infected cases, study subjects

Following informed consent, individuals with omicron BA.1 were co-enrolled into the ISARIC/WHO Clinical Characterisation Protocol for Severe Emerging Infections [Oxford REC C, reference 13/SC/0149] and the "Innate and adaptive immunity against SARS-CoV-2 in healthcare worker family and household members" protocol affiliated to the Gastro-intestinal illness in Oxford: COVID sub study [Sheffield REC, reference: 16/YH/0247] further approved by the University of Oxford Central University Research Ethics Committee. Diagnosis was confirmed through reporting of symptoms consistent with COVID-19 or a positive contact of a known Omicron case, and a test positive for SARS-CoV-2 using reverse transcriptase polymerase chain reaction (RT-PCR) from an upper respiratory tract (nose/throat) swab tested in accredited laboratories and lineage sequence confirmed through national reference laboratories. A blood sample was taken following consent at least 10 days after PCR test confirmation. Clinical information including severity of disease (mild, severe or critical infection according to recommendations from the World Health Organisation) and times between symptom onset and sampling and age of participant was captured for all individuals at the time of sampling.

Sera and PBMC from BA.2 infected cases, study subjects

Following informed consent, healthcare workers with BA.2 infection were co-enrolled under the Sheffield Biobank study (STHObs) (18/YH/0441) during March 2022 (median 22 March, BA.2 sequence was confirmed for all samples taken before 22 March). All individuals had PCR-confirmed symptomatic disease and sequence confirmed BA.2 infection through national UKHSA sequencing data. A blood sample was taken following consent at least 12 days after PCR test confirmation. Clinical information including

vaccination history, times between symptom onset and sampling and age of participant was captured for all individuals at the time of sampling.

Sera from BA.4/5 infected cases, study subjects

Following informed consent, individuals with omicron BA.4 or BA.5 were co-enrolled into one or more of the following three studies: the ISARIC/WHO Clinical Characterisation Protocol for Severe Emerging Infections [Oxford REC C, reference 13/SC/0149], the "Innate and adaptive immunity against SARS-CoV-2 in healthcare worker family and household members" protocol (approved by the University of Oxford Central University Research Ethics Committee), or the Gastro-intestinal illness in Oxford: COVID sub study [Sheffield REC, reference: 16/YH/0247]. Diagnosis was confirmed through reporting of symptoms consistent with COVID-19, hospital presentation, and a test positive for SARS-CoV-2 using reverse transcriptase polymerase chain reaction (RT-PCR) from an upper respiratory tract (nose/throat) swab tested in accredited laboratories and lineage sequence confirmed through national reference laboratories in the United Kingdom. A blood sample was taken following consent at least 14 days after PCR test confirmation. Clinical information including severity of disease (mild, severe or critical infection according to recommendations from the World Health Organisation) and times between symptom onset and sampling and age of participant was captured for all individuals at the time of sampling.

Sera from Pfizer vaccinees

Pfizer vaccine serum was obtained from volunteers who had received three doses of the BNT162b2 vaccine. Vaccinees were Health Care Workers, based at Oxford University Hospitals NHS Foundation Trust, not known to have prior infection with SARS-CoV-2 and were enrolled in the OPTIC Study as part of the Oxford Translational Gastrointestinal Unit GI Biobank Study 16/YH/0247 [research ethics committee (REC) at

Yorkshire & The Humber – Sheffield] which has been amended for this purpose on 8 June 2020. The study was conducted according to the principles of the Declaration of Helsinki (2008) and the International Conference on Harmonization (ICH) Good Clinical Practice (GCP) guidelines. Written informed consent was obtained for all participants enrolled in the study. Participants were sampled approximately 28 days (range 25-56) after receiving a third “booster dose of BNT162B2 vaccine. The mean age of vaccinees was 37 years (range 22-66), 21 male and 35 female.
