# Supplementary material for: Antigenic characterization of SARS-CoV-2 Omicron subvariant BA.4.6
Source: Cell Discov. 2022 Nov 29;8:127. doi: 10.1038/s41421-022-00493-0 (PMC9705283; doi:10.1038/s41421-022-00493-0)
Supplement: Supplementary file 2 — BA.4.6_Supplementary information [file 41421_2022_493_MOESM2_ESM.pdf]

Materials and Methods

Plasmid construction and pseudotyped lentiviral particles production

Pseudotyped lentivirus expressing SARS-CoV-2 S proteins for ancestral strains (Victoria, S247R), BA.1, BA.1.1, BA.2, BA.2.12.1, BA.2.75 and BA.4 were constructed as described before [1-4] with some modifications. A similar strategy was applied for to create BA.4.6 variant, where BA.4 was used as the template and two mutations were introduced: R346T and N658S. The construct was cloned by PCR amplification of vector and inserts, followed by Gibson assembly. To generate the insert fragments, the overlapping primers for individual variant were used separately to amplify, together with two primers of pcDNA3.1 vector (pcDNA3.1_BamHI_F and pcDNA3.1_Tag_S_EcoRI_R). The pcDNA3.1 vector was also amplified using pcDNA3.1_Tag_S_EcoRI_F and pcDNA3.1_BamHI_R primers. The primer pairs used in this study are shown in Supplementary Table S3. Construct was verified by Sanger sequencing after plasmid isolation using QIAGEN Miniprep kit (QIAGEN). The resulting S gene-carrying pcDNA3.1 was used for generating pseudoviral particles together with the lentiviral packaging vector and transfer vector encoding luciferase reporter.

Pseudoviral neutralization assay

The pseudoviral neutralization test were described previously [2] with some modifications. Briefly, four-fold serial dilutions of each mAb were incubated with pseudoviral particles at 37 °C, 5% CO_2 for 1 h. The stable HEK293T/17 cells expressing human ACE2 were then added to the mixture at 1.5 x 10^4 cells/well. At 48 h post transduction, culture supernatants were removed and 50 µL of 1:2 Bright-GloTM Luciferase assay system (Promega, USA) in 1x PBS was added into each well. The reaction was incubated at room temperature for 5 min and the firefly luciferase activity was measured using CLARIOstar (BMG Labtech, Ortenberg, Germany). The percentage of neutralization was calculated relative to the control. Probit analysis was used to estimate the value of dilution that inhibits half of the maximum pseudotyped lentivirus infection (PVNT50). To determine the neutralizing activity of vaccine sera, three-fold serial

dilutions of samples were incubated with pseudoviral particles for 1 hr and the same strategy as for mAbs was applied.

REFERENCES

1. Nie, J., et al., *Establishment and validation of a pseudovirus neutralization assay for SARS-CoV-2.* Emerg Microbes Infect, 2020. **9**(1): p. 680-686.
2. Liu, C., et al., *The antibody response to SARS-CoV-2 Beta underscores the antigenic distance to other variants.* Cell Host Microbe, 2022. **30**(1): p. 53-68 e12.
3. Nutalai, R., et al., *Potent cross-reactive antibodies following Omicron breakthrough in vaccinees.* Cell, 2022. **185**(12): p. 2116-2131 e18.
4. Tuekprakhon, A., et al., *Antibody escape of SARS-CoV-2 Omicron BA.4 and BA.5 from vaccine and BA.1 serum.* Cell, 2022. **185**(14): p. 2422-2433 e13.

Supplementary Table S1. Characteristics of participants

	BA.1 infection	BA.2 infection	BA.4/5 infection
**Participants**			
Female	7	19	6
Male	7	4	5
**Median Age (Y)**	22 (Range 21-56)	41 (Range 22-57)	42 (Range 20-94)
**Vaccine History**			
First dose	2020 Dec-2021 Jul	2020 Dec-2021 March	2020 Dec-2021 June
ChAdOx1	4	3	2
BNT162b2	9	20	8
J&J	1		
Second dose	2021 Jan-2021 Sep	2021 March-2021 May	2021 Feb-2021 Aug
ChAdOx1	4	3	2
BNT162b2	9	20	8
Third dose	2021 Sep-2022 Jan	2021 Sep-2021 Dec	2021 Oct-2021 Dec
BNT162b2	11	22	4
Moderna	0	1	4
**Infection History**	BA.1 (2021 Dec)	BA.2 (2022 Feb-2022 Apr)	BA.4/5 (2022 June)
Pre-1st dose			1
Pre-3rd dose	4		2
Post-vaccine (1 dose)	1		
Post-vaccine (2 doses)	2		
Post-vaccine (3 doses)	7	23	8
**Average days after infection (D)**	42 (Range 27-55)	28 (Range 12-43)	37 (Range 24-49)

Supplementary Table S2. IC50 ± SEM (µg/ml) values for Omicron mAbs and commercial monoclonals

a

mAbs	Victoria	BA.1	BA.1.1	BA.2	BA.4/5	BA.4.6
**Omi-02**	0.002 ± 0.001	0.004 ± 0.001	0.004 ± 0.001	0.003 ± 0.001	>10	>10
**Omi-03 (3-53)**	0.003 ± 0.000	0.005 ± 0.002	0.003 ± 0.001	0.008 ± 0.001	0.017 ± 0.005	0.006 ± 0.002
**Omi-06**	0.007 ± 0.000	0.017 ± 0.003	0.139 ± 0.033	0.039 ± 0.008	>10	>10
**Omi-08**	0.008 ± 0.004	0.003 ± 0.000	0.002 ± 0.000	0.114 ± 0.045	0.086 ± 0.005	0.033 ± 0.002
**Omi-09**	0.006 ± 0.002	0.005 ± 0.000	0.005 ± 0.002	0.008 ± 0.002	0.166 ± 0.007	0.108 ± 0.009
**Omi-12**	0.006 ± 0.002	0.002 ± 0.000	0.002 ± 0.001	0.003 ± 0.001	0.429 ± 0.060	0.074 ± 0.018
**Omi-16 (3-66)**	0.014 ± 0.003	0.012 ± 0.002	0.011 ± 0.003	0.034 ± 0.012	0.029 ± 0.007	0.007 ± 0.001
**Omi-17 (3-66)**	0.023 ± 0.011	0.018 ± 0.012	0.022 ± 0.009	0.060 ± 0.004	0.028 ± 0.001	0.039 ± 0.008
**Omi-18 (3-53)**	0.008 ± 0.003	0.002 ± 0.000	0.002 ± 0.000	0.005 ± 0.000	0.005 ± 0.001	0.006 ± 0.001
**Omi-20 (3-66)**	0.009 ± 0.002	0.006 ± 0.001	0.005 ± 0.001	0.015 ± 0.003	0.014 ± 0.006	0.008 ± 0.003
**Omi-23**	0.005 ± 0.002	0.029 ± 0.006	0.023 ± 0.12	0.019 ± 0.005	>10	>10
**Omi-24**	0.005 ±0.000	0.006 ± 0.002	0.054 ± 0.015	0.007 ± 0.001	>10	>10
**Omi-25**	0.005 ± 0.001	0.023 ± 0.005	0.027 ± 0.005	0.024 ± 0.004	>10	>10
**Omi-26**	0.002 ± 0.001	0.006 ± 0.002	0.005 ± 0.001	0.013 ± 0.001	>10	>10
**Omi-27 (3-66)**	0.008 ± 0.003	0.026 ± 0.006	0.034 ± 0.009	0.034 ± 0.005	0.069 ± 0.023	0.023 ± 0.002
**Omi-28 (3-66)**	0.022 ± 0.000	0.011 ± 0.004	0.009 ± 0.002	0.008 ± 0.000	0.028 ± 0.009	0.035 ± 0.011
**Omi-29 (3-53)**	0.014 ± 0.006	0.017 ± 0.003	0.016 ± 0.009	0.056 ± 0.014	0.396 ± 0.007	0.170 ± 0.030
**Omi-30**	0.012 ± 0.002	0.008 ± 0.003	0.008 ± 0.004	0.011 ± 0.002	>10	>10
**Omi-31**	0.376± 0.090	0.029± 0.002	0.031 ± 0.012	0.013 ± 0.002	>10	>10
**Omi-32**	0.010 ± 0.006	0.017 ± 0.000	>10	2.682 ± 0.553	0.035 ± 0.016	>10
**Omi-33**	0.027 ± 0.011	0.014 ± 0.005	0.042 ± 0.018	0.068 ± 0.022	0.013 ± 0.004	>10
**Omi-34**	0.007 ± 0.004	0.008 ± 0.001	0.062 ± 0.004	0.009 ± 0.003	>10	>10
**Omi-35**	0.018 ± 0.004	0.058 ± 0.006	0.381 ± 0.061	0.094 ± 0.004	1.687 ± 0.441	>10
**Omi-36 (3-66)**	0.022 ± 0.004	0.009 ± 0.003	0.009 ± 0.003	0.030 ± 0.014	0.024 ± 0.006	0.029 ± 0.001
**Omi-38**	0.015 ± 0.004	0.024 ± 0.015	>10	0.005 ± 0.000	0.005 ± 0.001	>10
**Omi-39**	0.014 ± 0.002	0.009 ± 0.004	>10	0.026 ± 0.011	0.035 ± 0.003	>10
**Omi-41**	>10	0.053 ± 0.028	0.037 ± 0.002	>10	>10	>10
**Omi-42**	0.013 ± 0.004	0.007 ± 0.004	0.006 ± 0.002	0.021 ± 0.011	0.013 ± 0.001	0.010 ± 0.001

b

	IC50 ± SEM (µg/mL)						
	Pseudovirus						
	Victoria	BA.1	BA.1.1	BA.2	BA.3	BA.4	BA.4.6
**AZD1061**	0.002 ± 0.001	0.308 ± 0.058	>10	0.008 ± 0.003	0.019 ± 0.007	0.015 ± 0.004	>10
**AZD8895**	0.001 ± 0.000	0.246 ± 0.027	0.100 ± 0.053	1.333 ± 0.317	>10	>10	>10
**AZD7442**	0.001 ± 0.000	0.232 ± 0.113	0.806 ± 0.093	0.008 ± 0.001	0.065 ± 0.011	0.065 ± 0.007	>10
**REGN10987**	0.002 ± 0.001	>10	>10	0.616 ± 0.347	>10	>10	>10
**REGN10933**	0.001 ± 0.002	>10	>10	>10	>10	>10	>10
**ADG10**	0.007 ± 0.002	>10	>10	>10	>10	>10	>10
**ADG20**	0.003 ± 0.002	0.348 ± 0.169	0.253 ± 0.070	>10	>10	>10	>10
**ADG30**	0.014 ± 0.006	>10	>10	>10	>10	>10	>10
**LY-CoV555**	0.002 ± 0.000	>10	>10	>10	>10	>10	>10
**LY-CoV016**	0.014 ± 0.010	>10	>10	>10	>10	>10	>10
**LY-CoV1404**	0.001 ± 0.000	0.002 ± 0.000	0.001 ± 0.000	0.001 ± 0.000	0.002 ± 0.000	0.002 ± 0.000	0.001 ± 0.000
**S309**	0.079 ± 0.027	0.113 ± 0.006	0.142 ± 0.012	0.638 ± 0.154	0.311 ± 0.023	0.689 ± 0.041	1.029 ± 0.058

Supplementary Table S3. Primer sequences used to generate pseudoviruses. Related to Plasmid construction and pseudotyped lentiviral particle production.

Primer	Sequence (5’to 3’)
pcDNA3.1_BamHI_F	GGATCCATGTTCCTGCTGACCACCAAGAG
pcDNA3.1_Tag_S_EcoRI_R	GAATTCTCACTTCTCGAACTGAGGGTGGC
pcDNA3.1_Tag_S_EcoRI_F	GCCACCCTCAGTTCGAGAAGTGAGAATTC
pcDNA3.1_BamHI_R	CTCTTGGTGGTCAGCAGGAACATGGATCC
BA.4+R346T_F	GTGTTCAATGCCACCACGTTCGCCAGCGTGTACG
BA.4+R346T_R	CGTACACGCTGGCGAACGTGGTGGCATTGAACAC
BA.4+N658S_F	CGGCGCCGAGTACGTGAATAGTAGCTACGAGTGCG
BA.4+N658S_R	CGCACTCGTAGCTACTATTCACGTACTCGGCGCCG
